# Optimizing Moss and Lichen Transplants as Biomonitors of Airborne Anthropogenic Microfibers

**DOI:** 10.3390/biology12101278

**Published:** 2023-09-25

**Authors:** Fiore Capozzi, Maria Cristina Sorrentino, Angelo Granata, Alessandro Vergara, Miriam Alberico, Manuela Rossi, Valeria Spagnuolo, Simonetta Giordano

**Affiliations:** 1 Department of Biology, University of Naples Federico II, 80126 Napoli, Italy; fiore.capozzi@unina.it (F.C.); mcristinasorrentino@gmail.com (M.C.S.); angelo.granata@unina.it (A.G.); giordano@unina.it (S.G.); 2 Department of Chemical Sciences, University of Naples Federico II, 80126 Napoles, Italy; alessandro.vergara@unina.it (A.V.); miriam.alberico@uniroma1.it (M.A.); 3 Department of Classics, University La Sapienza, 00185 Rome, Italy; 4 Department of Earth Sciences, University of Naples Federico II, 80126 Naples, Italy; manuela.rossi@unina.it

**Keywords:** *Hypnum cupressiforme*, *Pseudevernia furfuracea*, Raman microspectroscopy, microplastics, biomonitoring

## Abstract

**Simple Summary:**

Environmental pollution due to the presence of anthropogenic microfibers, including microplastics, is a problem affecting quality of life in modern society. Anthropogenic microfibers are ubiquitous, produced by a multitude of processes, and harmful to organisms and ecosystems. In this article, we present the results of an experiment aimed at optimizing the use of transplanted mosses and lichens as biomonitors (i.e., organisms capable of accumulating or reacting to the presence of pollutants) of anthropogenic microfibers. We found that the moss *H. cupressiforme* is preferable to the lichen *P. furfuracea*, especially when exposed without a covering net.

**Abstract:**

Anthropogenic microfibers (mfs) are synthetic particles composed of cellulose (cotton, rayon, acetate, etc.) or petrochemical-based polymers (i.e., microplastics—MPs) that are less than 5 mm in length. The accumulation of mfs, including MPs, in the moss *Hypnum cupressiforme* and the lichen *Pseudevernia furfuracea* was compared in a transplant experiment lasting 6 weeks. We also tested the effects of the bag used for transplants on the accumulation of mfs. Anthropogenic particles trapped by both biomonitors were mostly filamentous (99% mfs), and their number was overall higher in the moss (mean ± s.d. 102 ± 24) than in the lichen (mean ± s.d. 87 ± 17), at parity of sample weight. On average, mfs found in lichen were significantly longer than those found in moss bags, suggesting that lichens are less efficient at retaining smaller mfs. Exposure without the net yielded a higher mfs number accumulation in both species, indicating that “naked” transplants provide greater sensitivity. The calculation of daily fluxes evidenced a loss of mfs in the lichen, suggesting the presence of more stable bonds between moss and mfs. Raman microspectroscopy carried out on about 100 debris confirms the anthropogenic nature of mfs, of which 20% were MPs. Overall results indicate that moss is preferable to lichen in the biomonitoring of airborne mfs especially when exposed naked.

## 1. Introduction

Anthropogenic microfibers (mfs) are synthetic particles composed of cellulose (cotton, rayon, acetate, etc.) or petrochemical-based polymers (i.e., microplastics—MPs) that are less than 5 mm in length. These materials are a growing global concern given their widespread presence in every environmental compartment; nonetheless, to date most studies have focused on their assessment in marine and aquatic environments [1]. Plastics, because of their versatility and cheap costs, are used worldwide [2]; their employment in single use or short time objects contrasts with their very long degradation times (up to 1000 years) [3]. During this long presence in the environment, plastics break into pieces of various sizes and shapes including microplastics, and are being found and studied even in areas uninhabited by humans [4]. MPs such as polystyrene (PS) or polyvinyl chloride (PVC) can have mutagenic and carcinogenic toxic effects, but additionally, plastic debris could carry many inorganic and organic pollutants known to be mutagenic and carcinogenic to organisms following ingestion and inhalation [5]. It is well established that microplastics are present in the air [6], as well as in many consumer products such as foods and beverages [7], and even in human blood [8]. Therefore, it is necessary to monitor their presence in the environment.

In previous studies [9], we highlighted that the accumulation of airborne particulate matter (PM), to which mfs belong, by cryptogams is mainly based on passive mechanisms. As for plastics, we observed in a laboratory experiment, that polystyrene nanoparticles were uptaken in water cultures by the moss *Sphagnum palustre* L., and that post-exposure washing induced a loss of larger aggregates (>10 µm) [10]. More recently, in a study focused on the use of native mosses for the estimation of anthropogenic microfiber in terrestrial environments, Roblin and Aherne [11] adopted a methodology based on the visual identification of relatively large mfs (>30 µm, according to the authors’ data). The same approach was adopted by Jafarova et al. (2023) [12] to compare native mosses and lichens for the assessment of the atmospheric deposition of anthropogenic MPs. However, the uncertainty associated with the age of thalli is a limitation in the use of native species [13]. Recent literature indicates that transplants of cryptogams represent a promising option to evaluate the airborne deposition of MPs [14,15], also because transplants allow a defined exposure time. Nevertheless, as has already been investigated for other airborne pollutants, like metal(loid)s and PAHs, other methodological aspects should be considered to properly apply the transplant approach to mfs. A large body of literature demonstrated that in moss and lichen individual peculiar traits favor from time to time the accumulation/retention of pollutants as metal(loid)s and PAHs. However, very little is known about the interactions between mfs and plant biomonitors [10] and limited data are available comparing different biomonitors in the same context [12]. Considering that the visual identification of mfs can evidence only relatively large debris, the presence of the net used in transplants could substantially affect their accumulation.

Based on specific morphological traits of the two species, we predict that the moss *Hypnum cupressiforme* and the lichen *Pseudevernia furfuracea*, two widely employed biomonitors, behave differently in relation to anthropogenic microfibers accumulation when transplanted for the same exposure time. Further, the presence of the net used in transplants could substantially affect the accumulation of mfs. Therefore, we evaluated and compared in a transplant experiment (i) the mfs accumulation capacity of the two species exposed at the same sites; (ii) the effect of the bag on the uptake of mfs, exposing the biomonitors with and without a net. In addition, we used Raman microspectroscopy to identify the polymer type for a set of mfs [16].

## 2. Materials and Methods

### 2.1. Sampling

The species were collected in two semi-natural areas where they grow abundantly following the sampling protocol suggested by the International Cooperative Programme (ICP) on Vegetation [17]. Based on the results of a recent survey in semi-natural sites in the Campania region, southern Italy [18], the moss *Hypnum cupressiforme* Hedw. was collected from Mt. Taburno (MTB), which had the lowest mfs deposition among the sites in the survey (Lat. 41.102701°; Long 14.603371°; 1050 m. a.s.l.). Unfortunately, the test lichen *Pseudevernia furfuracea* (L.) Zopf. was scarcely present at the same collection site; therefore, it was collected from Mt. Matese (MTS, Lat. 41.468321°; Long 14.405094°; 1300 m. a.s.l.), a site similarly characterized by low mfs deposition, at least for moss.

### 2.2. Bag Preparation and Exposure

We manually cleaned the moss and lichen samples of extraneous materials, soil and other plant debris under a stereomicroscope while wearing nitrile gloves and cotton clothing, then dried them at 50 °C for 48 h. To avoid the possible release of MPs from the net, 1 g of sample for each species was exposed in triplicate in uncolored cotton bags (i.e., medical gauze 100% cotton with a surface of about 65 cm^2^) with a 2 mm mesh and a density of 15 mg thalli per cm^2^ bag surface [18]. Five samples of 1 g for each species were not exposed and analysed to evaluate the LOQ_T_ of the method.

**Figure 1:**
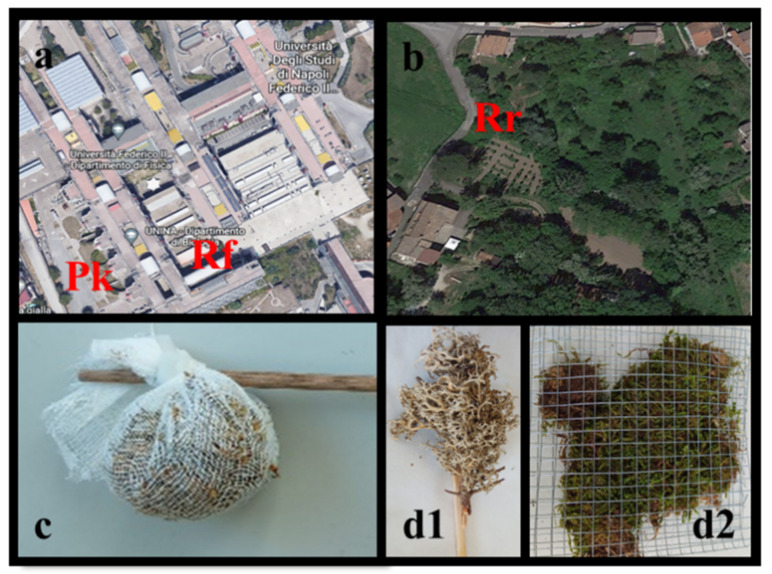
Site locations (**a**,**b**). Exposure in cotton bag (**c**) and naked ((**d1**) and (**d2**), for lichen and moss, respectively).

The transplants were exposed for 6 weeks, a time period well assessed for the monitoring of other air pollutants, during the autumn of 2021 (September–October), in three sites potentially characterized by different mf fallout: a rural site (Rr; 41°1′47.93″ N, 14°44′28.53″ E), a parking area (Pk; 40°50′14.42″ N, 14°11′0.79″ E), and the roof (Rf; 40°50′15.23″ N, 14°11′2.55″ E) of the Biology Department, the last two located in the University Campus of Monte Sant’Angelo in Naples (Figure 1a,b). To test the effects due to the bag on the uptake of mfs, a triplicate set of moss and lichen thalli was exposed without the bag at the roof site (Rf). Specifically, the lichen was exposed completely naked fixed to a wooden stick; for the moss, due to the impossibility of exposing it without a containing net, a metal grid with a 6 mm mesh was used to make up flat bags (Figure 1c,d).

### 2.3. Anthropogenic Microfiber Extraction

Pre-exposure and post-exposure 1 g plant samples were digested using the wet peroxide oxidation method described in Masura et al. (2015) [19] and Herrera et al. (2018) [20]. The samples were digested by adding 40 mL of 0.05 M Fe (II) solution per gram of moss or lichen sample, and then 40 mL of 35% H_2_O_2_; the mixture was left at room temperature for 5 min. The digestate was heated to 55 °C to boost the reaction, and further 20 mL aliquots of H_2_O_2_ were added in case of organic matter residuals. The samples were then vacuum filtered onto glass-fiber filter circles (MN GF-4, retention capacity: 1.4 µm), following Dris et al. (2016) [21] and dyed with 1 mL of Rose Bengal (4,5,6,7-tetrachloro-2′,4′,5′,7′-tetraiodofluorescein, 200 mg L^−1^), following Liebezeit et al. (2014) [22] and Kosuth et al. (2018) [23] to better evidence the organic matter. To estimate the number of mfs captured by the cotton net, it was processed similarly to the plant material (i.e., acid-digestion, filtration, and visual identification). The dyed filters were then transferred to glass petri dishes for storage and for assessment of mfs. Finally, the samples were labelled as follows: moss and lichen thalli (M_Th and L_Th) inside the bag, moss and lichen nets used for the bag preparation (M_N and L_N), the summation of the accumulated microplastics in the previous two, the whole bag (i.e., net plus thallus: M_All and L_All), and moss and lichen exposed without the bag, as explained in the previous paragraph (M_Naked and L_Naked). Throughout the sample collection, processing and analysis, procedural open-air blanks were used to determine potential contamination. Prior to use, water and peroxide blanks were vacuum filtered and observed as well to determine possible contamination by mfs.

### 2.4. Stereomicroscope Identification

A stereomicroscope (Leica Wild M8) was used for mfs visual identification following the criteria suggested by Sweden et al. (2007) [24] and Windsor et al. (2019) [25]. In the current study, to provide data comparable with previous studies by Roblin and Aherne (2020) [11], non-stained fibers that met at least two of the criteria were classified as anthropogenic mfs. The stereomicroscope was implemented with a micrometric grating lens for measuring the length of mfs.

### 2.5. Raman Microspectroscopy

Proof of the synthetic nature of the non-stained mfs can be efficiently provided by vibrational microspectroscopies such as Raman effect-based techniques [26,27]. A confocal Raman microscope (Jasco, Oklahoma City, OK, USA, NRS-3100) was used to obtain Raman spectra [28]. The 514 nm line of an air-cooled Ar^+^ laser (Melles Griot, Rochester, NY, USA, 35 LAP431 220) was injected into an integrated Olympus microscope and focused on isolated fibers with a laser spot diameter of approximately 1–5 μm. A 20×–100× objective with a final 3 mW power was used in the sample. A holographic edge filter was used to reject the excitation laser line. Raman backscattering was collected using a diffraction lattice of 1200 grooves/mm and 0.01 mm slit, corresponding to an average spectral resolution of up to 4 cm^−1^. Because of the fluorescence of the samples, the acquisition time was not the same for all. In some cases, a time of about 10–60 s was required to collect a complete dataset from a Peltier-cooled 1024 × 128 pixel CCD photon detector (Andor DU401BVI). Raman measurements were carried out in triplicate for the purpose of reproducibility for each spot sampled. Wavelength calibration was performed by using cyclohexane as a standard. Raman spectra were registered in the range of 200–3700 cm^−1^ for 106 randomly selected mfs samples. Inspection via optical microscopy allowed us to identify micrometric areas for Raman investigation.

### 2.6. Data Analysis

All data were processed using Microsoft Excel and IBM SPSS Statistics for Windows (IBM Corp. Released 2020, Version 27.0. Armonk, NY, USA). A Shapiro–Wilk’s test and Levene’s test were used to assess the normality and homogeneity of the variances of the datasets, respectively. The differences among means of different groups were assessed by ANOVA; in the case of *p* < 0.05 the post hoc Tukey’s test was followed for multiple comparisons.

The assessment of enrichment was conducted considering the limit of quantification of the technique (LOQ_T_) that was calculated as follows:

xN_i_ + 2*sN_i_

where xN_i_ is the mean number of mfs*g^−1^ in unexposed samples (n = 5), and sN_i_ is the corresponding standard deviation [29,30] (Couto et al., 2004 as modified in Ares et al., 2015). Only the samples whose accumulation exceeded the LOQ_T_ were considered to be significantly enriched in mfs.

The following formula developed by Capozzi et al. (2020) [9] was used to calculate the daily deposition flux:

ΘDF = N° of mfs/(S*d)

where ΘDF is the deposition flux [N° of mfs*m^−2^*d^−1^]; N° of mfs is the number of mfs accumulated (post minus pre-exposure content) in 1 g of plant tissue; S is the 2* SLA (specific leaf area), expressed as m^2^ g^−1^; d is the duration of exposure, expressed as days (42 in our experiment). For the calculation of fluxes, we used previously calculated SLAs for moss: 0.135 m^2^ g^−1^; and for lichen: 0.026 m^2^ g^−1^ [9]. The length class distribution was assessed by grouping mfs in five dimensional classes from 0 to 5 mm (step 1 mm), considering all mfs found in the different samples; the differences between the samples were analysed by Friedman ANOVA followed by a Wilcoxon matched pairs test.

## 3. Results

### 3.1. Number of Mfs in Moss and Lichen Transplants

**Figure 2:**
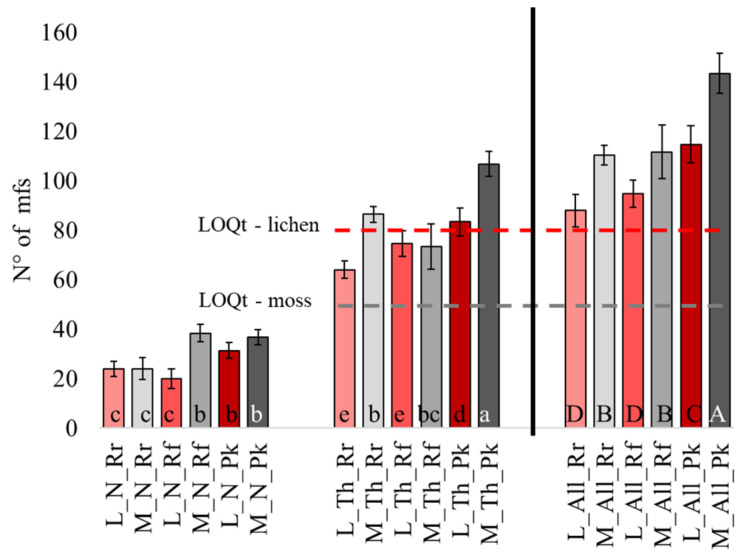
Number of mfs found in lichen (L), moss (M), cotton net (N), thalli (Th), and the sum of the latter two (N + Th = All) in the three test sites (Rr, Rf, Pk). The bar charts represent the average mfs number, and the error bars the standard deviation (n = 3). Different letters indicate significant differences according to Tukey’s test (*p* < 0.05). Further details are explained in the M&M section.

Most of the observed debris matching the visual criteria were microfibers (99%); therefore, these were counted, computed, and characterized. The average number of mfs in pre-exposed moss and lichen were, respectively, 38.2 ± 6.8 and 58.8 ± 12 (mean ± s.d., n = 5). To estimate the accumulation of mfs in the transplants, we considered the number of mfs exceeding the LOQ_T_ values (see method section), which were, respectively, 52 and 83 for moss and lichen. After exposure, the number of mfs was overall higher in the moss than in the lichen, with a range of 21–152 (mean ± s.d. 102 ± 24) and 60–120 (mean ± s.d. 87 ± 17), respectively. As for mfs accumulation in the thalli exposed in bags (M_Th and L_Th codes in Figure 2), all moss samples exceeded the LOQ_T_ value, whereas the exposed lichen exceeded the LOQ_T_ value only at the Pk site (Figure 2). Moreover, mf accumulation in the moss was comparable at Rr and Rf sites, and significantly higher at Pk; a similar trend was observed in the lichen considering mf content (i.e., with Rr = Rf significantly < Pk; Figure 2).

No mfs were observed in unexposed nets, while a noticeable entrapment of mfs was found in exposed nets, with a range of 20 (L_N at Rf) to 38 (M_N Rf and Pk), with the Pk site characterized by an overall higher mf deposition. By comparing biomonitors and relative nets, we saw that the number of mfs in the net was lower than in moss shoots at Rr and Pk and comparable in the case of Rf; in contrast, the number of mfs was always higher in the net than in the lichen thalli.

Summing up the mfs found in the thallus and in the net for both biomonitors (M_All and L_All codes in Figure 2), we recorded a higher number of mfs at the Pk site, whereas a comparable number of particles was found at Rr and Rf.

**Figure 3:**
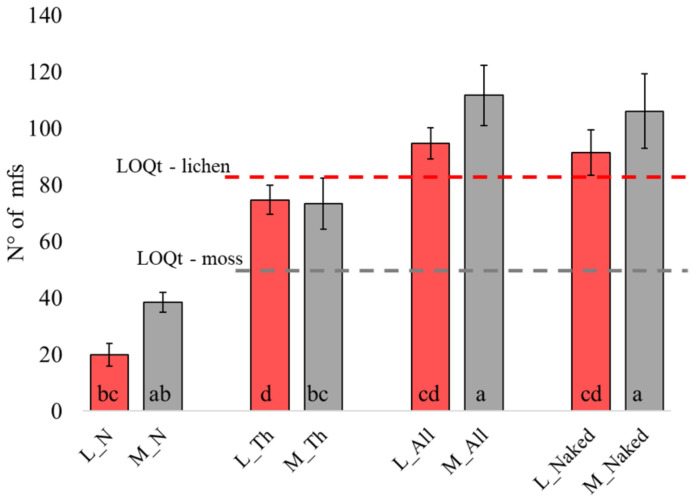
Number of mfs found in lichen (L), moss (M), cotton net (N), thalli (Th), the sum of latter two (N + Th = All), and in the two biomonitors exposed naked at Rf site. The bar charts represent the average mfs number and standard deviation (n = 3). Different letters indicate significant differences according to Tukey’s test (*p* < 0.05). Further details are explained in the M&M section.

In the parallel exposure on the roof of the Department (Rf) aimed at comparing “naked” vs. “in bag” exposure conditions, the lichen did not reach the LOQ_T_ when exposed inside a cotton bag, while the LOQ_T_ was crossed when the thalli were exposed naked. In the moss, mf accumulation exceeded the LOQ_T_ in both conditions, with a significantly higher accumulation when exposed naked. The exposure without the cotton net evidenced a mf accumulation like that obtained by summing the thallus and net contributions (Figure 3); in other words, the particles were no longer entrapped by the cotton net and could accumulate.

### 3.2. Length of Mfs in Moss and Lichen Transplants

The mfs found in lichen bags were on average longer than those found in moss bags, and comparable among the three exposure sites. The shortest mfs were observed in the moss exposed in the parking area (Pk) (Table 1).

**Figure 4:**
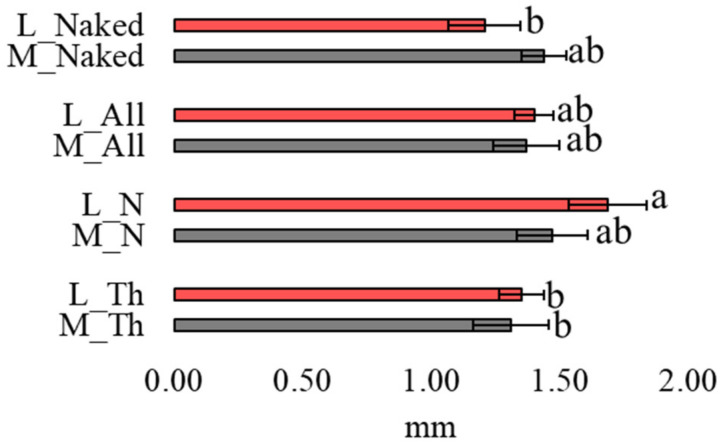
Length of mfs found in lichen (L), moss (M), cotton net (N), thalli (Th), the sum of latter two (N + Th = All) and in the two biomonitors exposed naked at Rf site. The bar charts represent the average microplastic lengths of the three means calculated on the total number of mfs found in each sample; error bars are standard deviations (for the total number of mfs for each sample, see Table S1). Different letters indicate significant differences according to Tukey’s test (*p* < 0.05). Further details are explained in the M&M section.

The fiber length ranged from 200 µm to 10.1 mm, but only fibers up to 5 mm (i.e., microplastics) were considered in the data analysis. Regarding the exposure condition (i.e., “naked” vs. “in bag” biomonitors), the average length of mfs was overall homogeneous in all samples except for the mfs entrapped on the net that were longer than those found on the thalli exposed within the bags; this difference was significant for the lichen (Figure 4).

Length class distribution of all mfs counted and measured in the different sites and exposure condition (Table 2) evidenced that, in both biomonitors, the most abundant dimensional classes were in the range 0–2 mm. The comparison between exposed moss and pre-exposed material showed a significant increment of mfs in all dimensional classes (even those not represented in pre-exposed samples, i.e., 3–5 mm), in all sites and conditions. By contrast, no significant difference was observed in exposed lichen where the shortest mfs decreased during the exposure.

### 3.3. Deposition Fluxes

The daily deposition fluxes calculated, based on the mfs accumulated by thalli, for the two biomonitors and in the different exposure conditions, are reported in Table 3. In the lichen thallus, deposition fluxes were characterized by a loss of mfs at Rr and Rf while a positive flux was recorded in the parking area (Pk) when they were exposed in bags. Instead, the exposure in naked conditions at Rf indicated a positive flux of 9.5 mfs m^2^ d^−1^. For the moss exposed in bags, the fluxes were always positive with the highest value recorded at Pk of 9.5 mfs m^2^ d^−1^. The naked moss exposed at Rf showed a positive flux of 9.3 mfs m^2^ d^−1^, higher than the flux calculated for the moss exposed inside the cotton bag at the same site (3.6 mfs m^2^ d^−1^) and consistent with the flux provided by the naked lichen.

### 3.4. Raman Analyses

**Figure 5:**
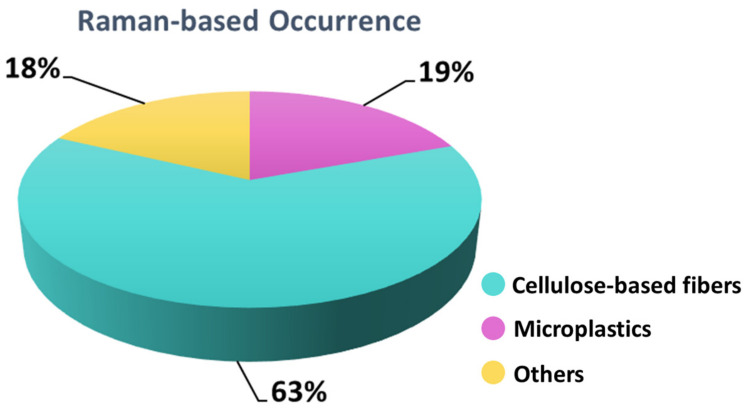
Distribution of microplastics and cellulose-based fibers identified. Others: compounds not included in the classification of “cellulose-based fibers” and “microplastics”.

**Figure 6:**
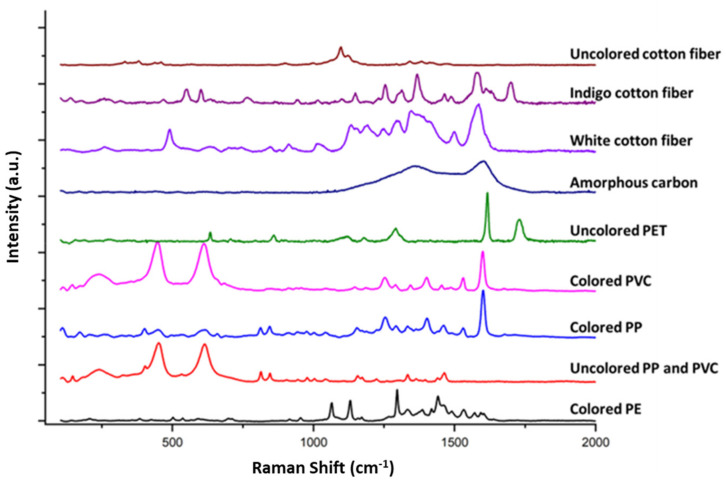
Representative collected Raman spectra, from top to bottom, of cellulose-based fibers, amorphous carbon and microplastics. Spectra were collected using 514 nm laser line, 2 mW power at the sample.

Raman spectra were registered to identify the chemical nature of the materials under investigation. Out of the 106 samples, 39 were fluorescent at the laser line used for the measurement, while 67 provided Raman spectra that were clustered in classes according to the observed Raman features (see Table 4 for literature with band assignment). The overall quantitative distribution of the identified microplastics and microfibers is summarized in Figure 5. Representative collected Raman spectra are also reported in Figure 6. Raman assignment was based on the acquisition of microplastic reference Raman spectra collected from a polymer kit purchased from the Center for Marine Debris Research, together with specific literature on microplastic and microfibers (cited in Table 4).

The spectral range of these polymers covers all spectral features from 200 cm^−1^ to 3700 cm^−1^. This spectral range covers the fingerprint area and the CH stretching modes of alkyls, alkenes, and aromatic protons [26]. Investigated samples showed features compatible with both plastics of common use, carbon rubber, carbon-based materials, and some textiles (Table 4).

## 4. Discussion

This is the first contribution focused on the methodological aspects of the use of moss and lichen in a transplant experiment for the assessment of atmospheric mfs deposition. Our data evidenced that the moss showed a higher accumulation of mfs, since, at parity of weight exposed and time frame, its number exceeded the threshold of the LOQ_T_ set to underline significant accumulation. In contrast, mfs counted on the lichen thalli must be summed to those found on the bag net to reach a significant accumulation. In any case, both biomonitors gave consistent information, highlighting a greater number of mfs deposited in the parking area, followed by the rural area and the roof of the Department.

In both species, the parallel exposure with and without a bag showed that the 2 mm net blocked a considerable fraction of mfs, which could not reach the thalli; as a proof of this result, the thalli exposed naked had an mf content statistically comparable with that deriving from the summation of mfs entrapped by both the net and the thallus. This result suggests that the naked exposure is more effective at increasing the sensitivity of the method. The consistent pattern shown by the two biomonitors in this case is not a constant rule, since it depends on the target pollutant; indeed, for PAHs, the moss and lichen behave differently, since the moss mainly accumulates high molecular weight PAHs, largely linked to PM, while the lichen also entraps low molecular weight PAHs, mostly gaseous at an environmental temperature, into the multi-layered thallus [9]. Anthropogenic mfs are quite large (up to 5 mm) and it is reasonable to assume that they mainly accumulate by adhesion to the surface of the biomonitor; therefore, at parity of weight, the higher the exposed surface is (i.e., the moss, in which the exposed surface per gram is one order of magnitude higher than the lichen), the higher the accumulation is. Because the calculation of daily fluxes is normalized to the species surface, a similar flux was detected in the two species. Even if this work is not intended as a biomonitoring survey, but is focused on the methodology, the discovery of a greater number of mfs in both biomonitors in the parking area, where there is an expected source of emission, is further proof of the feasibility of this methodology. Consistent with the accumulation data, the fluxes were also greater in the parking area; here in fact, the continuous movement of vehicles, mostly cars and buses, likely leads to the release of mfs from the friction of the tires on the asphalt [35]. Recent studies using transplants of the lichen *Evernia prunastri* evidenced a clear increasing gradient from the city center (44 ± 1 nr. mfs g^−1^ dw) of Milan (N. Italy) towards the periphery (56 ± 5 nr. mfs g^−1^ dw) [14]. Similarly, Bertrim and Aherne (2023) [15], found a mfs accumulation significantly increased with urban intensity (from 13 to 29 mfs m^−2^ day^−1^) in southern Ontario (Canada).

Regarding fluxes, appropriate comparisons are not feasible with published works. The few articles focused on native and transplanted cryptogams as biomonitors of mfs calculate the deposition flux as the number of mfs per square meter of lichen or moss carpet [11,36] or per surface of the bag [15] or as the mass/area ratio of the thallus [14]. Due to the indetermination of the age of the native material, the rough approximation of the surface useful for the uptake, and the different equation to express the deposition fluxes, any direct comparison with the already published data is qualitative and should be considered with caution. In an attempt to make such a comparison, in our sites we observed depositions relatively lower compared to those reported by Bertrim and Aherne (2023) [15] and Jafarova et al. (2022) [14] who indicated, respectively, a daily deposition of MPs ranging from 21 to 60 and from 43 to 119 MPs m^−2^ d^−1^ from low-density to densely populated areas.

In both species, the most abundant dimensional mf classes were in the range 0–2 mm as previously observed in native mosses by Roblin and Aherne (2020) [11] and Capozzi et al. (2023) [37]. Anthropogenic mfs accumulated by the lichen were on average longer than those observed on the moss. This could be explained by considering the thallus morphology: in fact, lichen thallus has a smooth and even surface, while that of the moss is more irregular, likely allowing a greater accumulation of smaller particles [38]. More generally, the compact structure, with a quite flat surface of the lichen, probably allows a greater accumulation of longer mfs more susceptible to be lost after rain or wind; this may account for the loss of mfs during exposure, which is reflected in the signal provided by the lichen. On the contrary, the loose and articulated architecture of the moss, with spaces between shoots, branches and leaves probably allows it to trap and retain mfs more effectively, with a higher accumulation.

Comparing the distribution of mf length classes in pre- and post-exposure samples, we can hypothesize that mfs in moss add up to those already present in the pre-exposure material (an increase in mfs was observed in all size classes). In the lichen in contrast, despite the loss of the smallest mfs, the frequency of the dimensional classes did not change significantly; this result is further evidence of a different capacity of adhesion and retention of mfs by the two species.

Interestingly, the net entrapped the longest mfs; this supports the hypothesis that the 2-mm-sized net sequesters, on average, the longer particles while letting the shorter ones pass.

Finally, although further studies are needed to understand the retention capacity of mfs by moss and lichen, as well as the mechanisms possibly determining the adhesion of mfs to thalli, the results of the present work are based on mfs adsorbed to thallus, therefore, adhering to it with a merely passive mechanism. This does not exclude the possibility that very small mfs and nanoplastics (i.e., particles that cannot be identified by visual inspection), can enter the cells, like other nanomaterials. It is reported that mfs can adhere to organic matter through H-bonds, Van der Waals forces and π interactions, and other weak bonds [39]. In addition, it must be considered that mfs, at least in a water environment, undergo real colonization processes by microorganisms, with the consequent formation of biofilms (also called eco-corona [40]), which could increase their ability to adhere to biomonitors. Moreover, we should consider the different composition of the cell wall of moss (cellulosic) and lichen (chitin of fungi and associated exudates of the photobiont [41]), which could broaden the range of possible chemical bonds, as well as lead to different binding capacities both on a physical-mechanical and chemical basis.

The Raman analysis proved the presence of a high number of anthropogenic micro fibers dominated by cellulose-based fibers together with about 20% of microplastics, a proportion in line with the observation by Stanton et al., 2019 [42]. Our data are also consistent with Roblin and Aherne (2020), who reported a proportion of MPs in the range of 13–27% in samples collected from remote areas; on the other hand, Loppi et al. (2021) [36], near a landfill dumping site located in Italy, observed a proportion of about 40% MPs with the remaining fibers belonging to different natural textiles.

## 5. Conclusions

Transplanted *H. cupressiforme* and *P. furfuracea* showed a good ability for the estimation of atmospheric deposition of mfs even in the short period of exposure of 6 weeks.

*H. cupressiforme*, with a considerably higher surface to mass ratio, showed a greater accumulation capacity. The 2 mm net is an obstacle to the accumulation of microplastics on the thalli, acting as a particle sequestrator, especially for mfs of a higher dimension; therefore, a naked exposure (or a wider mesh bag, e.g., 6 mm) should be preferred additionally to increase the sensitivity of the method (i.e., higher accumulation with consequent higher signal). The fact that the fluxes in the moss and lichen are comparable indicates that the accumulation of mfs is limited to their surface. The loss of mfs observed only in lichen suggests a lower capacity to retain mfs than moss, which therefore should be preferred in the biomonitoring of airborne mfs. Future research to improve the method should be implemented by testing different exposure times, species retention capacity including higher plants, as well as mf accumulation in indoor and work environments.

## Figures and Tables

**Table 1:** Average length (mm) of mfs and standard deviation. M_All and L_All: sum of mfs found on net plus thallus for moss (M) and lichen (L).

Sample	Site	n.	mean	s.d.	Tukey’s
L_All	Rr	3	1.50	0.03	a *
L_All	Pk	3	1.42	0.07	ab
L_All	Rf	3	1.40	0.08	ab
M_All	Rr	3	1.26	0.06	b
M_All	Pk	3	1.08	0.04	c
M_All	Rf	3	1.37	0.02	ab

* Different letters indicate significant differences according to Tukey’s post hoc test (*p* < 0.05).

**Table 2:** Difference in mfs size distribution divided in 5 dimensional classes from 0 to 5 mm. The values indicate the frequencies of each class.

		M_Pre-expo	M_Rr	M_Pk	M_Rf	M_Rf_Naked	L_Pre-expo	L_Rr	L_Pk	L_Rf	L_Rf_Naked
**A**	0 < mfs < 1	**72**	118	197	108	117	**141**	77	108	95	112
**B**	1 ≤ mfs < 2	**83**	158	191	181	109	**103**	117	165	123	110
**C**	2 ≤ mfs < 3	**15**	46	38	50	76	**39**	54	51	51	29
**D**	3 ≤ mfs < 4	**0**	7	3	8	12	**8**	11	6	10	11
**E**	4 ≤ mfs ≤ 5	**0**	2	0	2	2	**2**	5	3	4	0
		b	a	a	a	a	n.s.	n.s.	n.s.	n.s.	n.s.
**Friedman ANOVA**			Chi Sqr. (N = 5, df = 4) = 10; *p* = 0.04					Chi Sqr. (N = 5, df = 4) = 2.5; *p* = 0.65			

Different letters indicate significant difference at *p* < 0.05 according to Wilcoxon Matched Pairs Test; n.s. = not significant.

**Table 3:** Deposition fluxes (N° of mfs m^−2^ d^−2^) calculated in the thalli (Th) of the two biomonitors exposed in bags and exposed naked at the three test sites (see M&M section for further details).

Biomonitor	Site	Test Portion	Mean mfs m^2^ d^−1^	s.d.
L	Rr	Th	−15.6	3.3
L	Pk	Th	2.1	5.2
L	Rf	Th	−5.8	4.7
L	Rf	Naked	9.5	7.4
M	Rr	Th	5.9	0.6
M	Pk	Th	9.5	0.9
M	Rf	Th	3.6	1.6
M	Rf	Naked	9.3	2.3

**Table 4:** Raman shifts and assignment of microplastic and fiber classes. A laser line 514 nm, 2 mW at the sample was used. Others: compounds not included in the classification of “cellulose-based fibers” and “microplastics”. Most valuable spectroscopic markers are in bold.

Class	Number of Samples	Raman Shift (cm^−1^)	Raman Assignment	Reference
Microplastics	3	**1065**, **1131**, 1171, **1297**, 1334, 1386, 1418, **1441**, 2724, **2849**, **2883**	Colored PE	Anger et al., (2018) [26]
Microplastics	3	404, **452**, 532, **615**, 814, 846, 944, 977, 1003, 1042, 1157, 1225, 1334, 1440, 1464, 2725, **2845**, **2888**, 2909, 2929, **2957**	Uncolored PP and PVC	Chakraborty et al., (2022) [27]
Microplastics	4	446, 617, 812, 844, 944, 976, 1000, 1040, 1154, **1253**, 1334, **1402**, 1461, 1530, 1601, **2843**, **2887**, 2908, 2927, **2956**	Colored PP	Anger et al., (2018) [26]
Microplastics	1	**447**, **611**, 1145, 1184, 1254, 1291, 1343, 1401, 1455, 1530, 1600	Colored PVC	Chakraborty et al., (2022) [27]
Microplastics	2	634, 859, 1118, 1178, 1292, **1617**, **1731**, 2962, 3082	Uncolored PET	Anger et al., (2018) [26]
Other	12	**1367**, **1601**	Amorphous carbon	Li et al., (2023) [31]
Cellulose based fibers	24	491, 1016, 1134, **1149**, 1192, 1249, 1294, **1347**, 1375, 1409, 1500, **1586**	Grey/white dyed cotton fiber	Zapata et al., (2022) [32]
Cellulose based fibers	16	258, 468, **553**, **601**, 766, 945, 1016, 1099, 1149, **1254**, 1313, **1368**, 1464, **1576**, 1611, **1700**	Indigo dyed cotton fiber	Karapanayiotis et al., (2004) [33]
Cellulose based fibers	2	332, 381, 437, 461, 897, 1000, 1098, 1121, 1149, 1342, 1381, 1413	Uncolored cotton fiber	Was-Gubala and Machnowski, (2014) [34]

## Data Availability

All data are reported in the text.

## Supplementary Materials

The following supporting information can be downloaded at: https://www.mdpi.com/article/10.3390/biology12101278/s1, Table S1—N° of anthropogenic microfibers and relative lengths in POST-EXPOSURE and PRE-EXPOSURE moss and lichen.
