# Supplementary material for: Optimizing Moss and Lichen Transplants as Biomonitors of Airborne Anthropogenic Microfibers
Source: Biology (Basel). 2023 Sep 25;12(10):1278. doi: 10.3390/biology12101278 (PMC10604676; doi:10.3390/biology12101278)
Supplement: Supplementary file 1 [file biology-12-01278-s001.zip › biology-2553843-supplementary.pdf]

Table S1 - N° of anthropogenic microfibers and relative lengths in POST-EXPOSURE and PRE-EXPOSURE moss and lichen

Biomonitor	Site	Bag portion	N of mfs	Average Length	Median±MAD - N of mfs	Median±MAD - Lenght
POST EXPOSURE						
L	Rr	Th	60	1.34	65±2	1.4±0.0
L	Rr	Th	65	1.39		
L	Rr	Th	67	1.41		
L	Rr	N	21	1.85	24±2	1.8±0.0
L	Rr	N	24	1.86		
L	Rr	N	27	1.76		
L	Rr	All	81	1.47	89±4	1.5±0.0
L	Rr	All	89	1.52		
L	Rr	All	94	1.51		
L	Pk	Th	77	1.48	85±4	1.3±0.1
L	Pk	Th	85	1.22		
L	Pk	Th	88	1.34		
L	Pk	N	29	1.56	30±2	1.6±0.1
L	Pk	N	35	1.71		
L	Pk	N	30	1.54		
L	Pk	All	106	1.50	118±5	1.4±0.1
L	Pk	All	120	1.36		
L	Pk	All	118	1.39		
L	Rf	Th	69	1.44	76±3	1.3±0.1
L	Rf	Th	79	1.29		
L	Rf	Th	76	1.26		
L	Rf	N	20	1.61	20±3	1.6±0.1
L	Rf	N	16	1.46		
L	Rf	N	24	1.84		
L	Rf	All	89	1.48	95±4	1.4±0.1
L	Rf	All	95	1.32		
L	Rf	All	100	1.40		
M	Rr	Th	85	1.29	85±2	1.3±0.0
M	Rr	Th	84	1.22		
M	Rr	Th	90	1.28		
M	Rr	N	29	1.42	22±3	1.41±0.11
M	Rr	N	22	1.41		
M	Rr	N	21	1.09		
M	Rr	All	114	1.32	111±3	1.3±0.1
M	Rr	All	106	1.26		
M	Rr	All	111	1.21		
M	Pk	Th	102	1.02	106±3	1.0±0.0
M	Pk	Th	112	1.03		
M	Pk	Th	106	0.97		
M	Pk	N	34	1.26	36±2	1.3±0.0
M	Pk	N	40	1.34		
M	Pk	N	36	1.26		
M	Pk	All	136	1.09	142±5	1.1±0.0
M	Pk	All	152	1.11		
M	Pk	All	142	1.04		
M	Rf	Th	65	1.30	72±6	1.3±0.0
M	Rf	Th	72	1.31		
M	Rf	Th	83	1.33		
M	Rf	N	35	1.53	38±2	1.5±0.1
M	Rf	N	42	1.40		
M	Rf	N	38	1.49		
M	Rf	All	100	1.38	114±7	1.4±0.0
M	Rf	All	114	1.34		
M	Rf	All	121	1.38		
PRE EXPOSURE						
M			45	1.12	37±4.8	1.12±0.104
M			28	1.02		
M			37	1.07		
M			37	1.22		
M			44	1.39		
L			78	1.14	58±8	1.24±0.076
L			58	1.12		
L			60	1.35		
L			47	1.29		
L			51	1.24		

Biomonitor	Bag portion	N of mfs	Average Length	Median±MAD - N of mfs	Median±MAD - Lenght
POST EXPOSURE					
L	Th	69	1.44	76±3	1.3±0.1
L	Th	79	1.29		
L	Th	76	1.26		
L	N	20	1.61	20±23	1.6±0.1
L	N	16	1.46		
L	N	24	1.84		
L	All	89	1.48	95±4	1.4±0.1
L	All	95	1.32		
L	All	100	1.40		
L	Naked	84	1.09	90±5	1.2±0.1
L	Naked	100	1.36		
L	Naked	90	1.17		
M	Th	65	1.30	72±6	1.3±0.0
M	Th	72	1.31		
M	Th	83	1.33		
M	N	35	1.53	38±2	1.5±0.1
M	N	42	1.40		
M	N	38	1.49		
M	All	100	1.38	114±7	1.4±0.1
M	All	114	1.34		
M	All	121	1.38		
M	Naked	94	1.34	104±9	1.5±0.1
M	Naked	120	1.51		
M	Naked	104	1.46		
